# A cell competition system with one gene expression from a single-copy gene in one cell

**DOI:** 10.1371/journal.pone.0302451

**Published:** 2024-07-05

**Authors:** Yoshinori Hasegawa, Megumi Nakano, Tsutomu Hosouchi, Takashi Watanabe, Izumi Yamaguchi, Manabu Nakayama, Osamu Ohara

**Affiliations:** 1 Department of Applied Genomics, Kazusa DNA Research Institute, Kisarazu, Chiba, Japan; 2 Kazusa Genome Technologies Inc., Kisarazu, Chiba, Japan; 3 Department of Frontier Research and Development, Kazusa DNA Research Institute, Kisarazu, Chiba, Japan; National Institute of Cancer Research, TAIWAN

## Abstract

Even with advanced plasmid and viral vectors, attaining copy numbers of multiple genes among different transfected cells is challenging. We achieved one gene expression from a single-copy gene in one cell using a transgene competition system, a combination of the Kazusa cDNA clones and our dual recombinase-mediated cassette exchange system. All 48 nuclear receptors were simultaneously expressed in one dish at the same expression level in HEK293 using this system, and the cell proliferation rate was compared. Significant differences were observed between cells transfected with CMV- or EF1 promoter-driven expression of the 48 nuclear receptors after 8 weeks. The EF1-NR1I2 cell line, which exhibited the highest increase from 2 to 8 weeks, showed 1.13-fold higher proliferation than the EF1-DsRed line. On the other hand, the EF1-NR4A1 cell line, which showed the maximum decrease at 8 weeks, showed 0.88-fold lower proliferation than the EF1-DsRed line. The results were confirmed in both our transgene competition system and long-term growth experiments. Our transgene competition system offers a wide-range, simple, and accurate cell competition method.

## Introduction

Cell competition (CC) is a biological mechanism highly conserved from *Drosophila* to vertebrates and results in the elimination of less fit cells by their more fit neighbors. CC is also involved in the interactions between tumor and neighbor cells, as they are crucial to the initial events of tissue transformation and subsequent tumor outgrowth and invasion [1,2]. In particular, overexpression or activation of an oncogene frequently leads to uncontrolled cell growth or growth arrest [3–5]. Even the overexpression of a normal gene could influence the pathology of cell dysfunction, diabetes, and programmed cell death [6,7].

Gene function screening is widely conducted using plasmid and viral vectors [8–11]. Although both vectors are convenient to use, stable gene expression with these two vector systems is associated with uncontrollable copy numbers in the target cell line, positional effect expression, and disruption of genes at the insertion site. For reproducible and competitive functional expression of the introduced transgenes, the expression of all transgenes should be stable and at similar levels. To address these issues, we applied a dual recombinase-mediated cassette exchange (RMCE) system, which is an efficient gene insertion system using the VCre/VloxP and Scre/SloxP recombination systems [12]. The two systems, VCre/VloxP and SCre/SloxP, developed by our group, do not cross-react with the Cre/loxP and Flp/FRT systems and represent novel site-specific recombination systems [13]. Dual RMCE is performed using two recombinases with distinct target specificities, demonstrating remarkably higher efficiency than RMCE performed using a single site-specific DNA recombinase [14]. With our system, the efficiency of gene insertion in the plasmid-transfected cells reached 20%. By utilizing this system and Kazusa cDNA clones, multiple genes could be expressed simultaneously in a cell culture dish, in which only one gene from a single copy of plasmid is expressed in one cell.

In this study, to confirm its effectiveness toward CC screening, all 48 members of the nuclear receptor (NR) family were overexpressed in HEK293 under CMV or EF1 promoters, and a cell proliferation assay was performed during long-term cell culture. Nuclear receptors have been implicated in the etiology of various diseases; they are also associated with cancer [15]. NRs are involved in kidney cancer, and aberrant expression of NRs in renal clear cell carcinoma has been reported [16,17]. Furthermore, for the sake of convenience, Slox and Vlox target sequences for dual RMCE were added on both sides of the NRs using two-step PCR. This new transgene competition system, combined with the 10,000 comprehensive KDRI ORF clones [8,18] and HaloTag clones [19], offers a wide-range, simple, and accurate CC method.

## Materials and methods

### Cell culture and cell line production

The HEK293 cell line was obtained from JCRB Cell Bank and was cultured in Dulbecco’s modified Eagle’s medium (043–30085; Fujifilm Wako, Osaka, Japan) supplemented with 10% fetal bovine serum at 37°C in the presence of 5% CO_2_. A single copy of a previously reported CMV- or EF1 promoter-driven Vlox-Slox cassette [12] was introduced into the AAVS Safe Harbor site using the AAVS-TALEN Safe Harbor Gene Editing Kit (Transposagen Biopharmaceuticals, Lexington, KY). The transfects were selected using 1 μg/mL puromycin (A1113803; Thermo Fisher Scientific, Waltham, MA) with a limited dilution.

### Construction of NR48 genes and performance of dual RMCE

**Fig 1:**
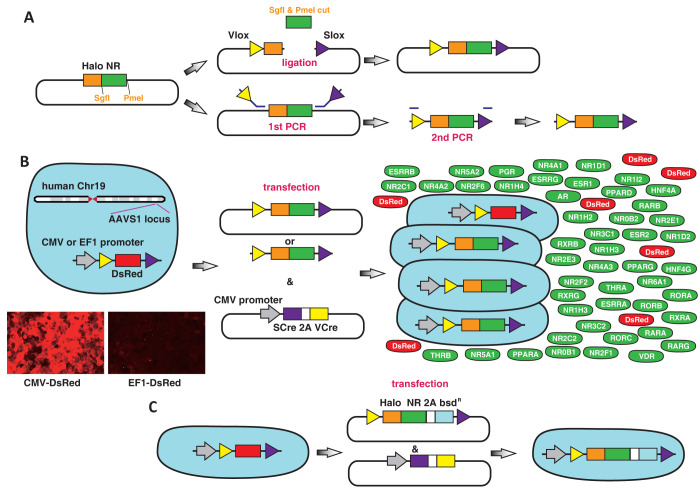
Expression of nuclear receptor (NR) genes using dual recombinase-mediated cassette exchange. A. HaloTag 48 NR genes were inserted between Vlox and Slox sites in HEK293 using two methods: Plasmid construction with restriction enzyme digestion and a PCR-based method. B. The NR genes sandwiched between the Vlox and Slox sites were transfected into HEK293 cells using the SCre-VCre recombinase plasmid. The photograph shows CMV- or EF1 promoter-driven DsRed expression in HEK293 cells. C. Stably transfected cell lines were used for confirmation of gene expression using the NR48 screening experiments; a blasticidin resistance gene was fused to Halo-NRs.

Promoterless Kazusa Flexi HaloTag clones of the 48 NRs (S1 Table) were cut with SgfI and PmeI restriction enzymes and inserted into another promoterless plasmid with Vlox and Slox sequences (Fig 1A). Equal amounts of the 48 NR plasmids were mixed. For convenience, Slox and Vlox sequences were added to the HaloTag Flexi KDRI NR clones using PCR (Fig 1A). After removing excess primers and PCR product contaminants, the PCR products were mixed at equimolar proportions. Dual RMCE reactions were performed following the methods described by Hasegawa et al. [12]. Two micrograms of the plasmid mixture of the 48 NRs or PCR products were cotransfected with 0.4 μg recombinase plasmids into 80% confluent cells plated in 6-well plates using Trans-IT 293 (MIR2704; Mirus, Madison, WI) following the manufacturer’s instructions (Fig 1B). The transfected cells were passaged every 2–3 days when the cultures reached 80%–90% confluency.

### EF1-NR4A1, EF1-NR1I2, and EF1-RORA expressing stable cell line production

In order to confirm the results of transgene competition, EF1-NR1I2, EF1-NR4A1, and EF1-RORA expressing stable cell lines were prepared using the NR48 screening experiment constructs carrying the 2A-blasticidin resistance gene fused to Halo-NRs (Fig 1C). Two micrograms of these plasmids were transfected with 0.4 μg of recombinase plasmids, and the transfected HEK293 cells were selected using 5 μg/mL blasticidin S (A1113903; Thermo) with limited dilution. Cell proliferation of each cell line (six lines of EF1-NR1I2, six lines of EF1-NR4A1, six lines of EF1-RORA, and three replicated lines of EF1-DsRed) was assessed using the Celltiter-Glo 2.0 luminescent cell viability assay kit (G9241; Promega, Madison, MA). In order to confirm the results of the luminescent cell viability assay, we also conducted cell counting using a Countess Automated Cell Counter (Thermo). The cells were seeded in a 10-cm dish at 5 × 10^5^ cells and counted after 63 h.

### Total RNA extraction, library preparation, and next-generation sequencing (NGS)

Total RNA was collected from the cells of the competition experiments at 2-, 4-, 6-, and 8-weeks post-transfection and 18 stable cell lines at 5-weeks post-transfection. The RNA was extracted from 50% confluent cells on a 10 cm-dish using TRIzol reagent (15596026; Thermo), as described previously [12]. The concentration and quality of the RNA were verified using a Qubit fluorometer (Thermo) and Agilent 2100 bioanalyzer, respectively. Two hundred nanograms of a purified aliquot of the total RNA was used for whole RNA-Seq library preparation at 2-, 4-, 6-, and 8-weeks post-transfection using the NEBNext Single Cell/Low Input RNA Library Prep Kit for Illumina (E6420; NEB, Beverly, MA), following the manufacturer’s instructions. Briefly, cDNA was amplified with seven cycles of PCR, and then adaptor-ligated DNA was amplified with five cycles of PCR. HaloTag 48 NR genes were concentrated using two-step PCR after amplifying cDNA with the NEBNext Single Cell/Low Input RNA Library Prep Kit for Illumina from 50 ng total RNA. The amplified HaloTag 48 NR DNA (100 ng) was used for library preparation using the NEBNext Ultra II FS DNA Library Prep Kit for Illumina (E7805; NEB). Details of primers and PCR conditions are presented in S2 Table. The libraries of whole RNA-Seq of 18 stable cell lines were prepared from 1000 ng total RNA using the NEBNext Ultra II Directional RNA Library Prep Kit for Illumina (E7760; NEB) according to the manufacturer’s instructions. The whole RNA-Seq and HaloTag 48 NR libraries were sequenced on a NextSeq 500 system using 75 bp single-end reads.

### RNA-Seq data analysis

All analyses were performed using the CLC genomics workbench version 23 (QIAGEN, Hilden, Germany) with the default settings. Using the”Trim Reads” tool, adapter sequences were removed from the raw reads, and base trimming was conducted. Reads shorter than 25 bp were removed prior to mapping. Each read was mapped to the human genome hg38 using the”RNA-Seq Analysis” tool. For concentrated HaloTag 48 NR genes, the reads were mapped to the sequences of the 48 NR genes listed in S1 Table.

## Results

### Plasmid gene insertion

**Fig 2:**
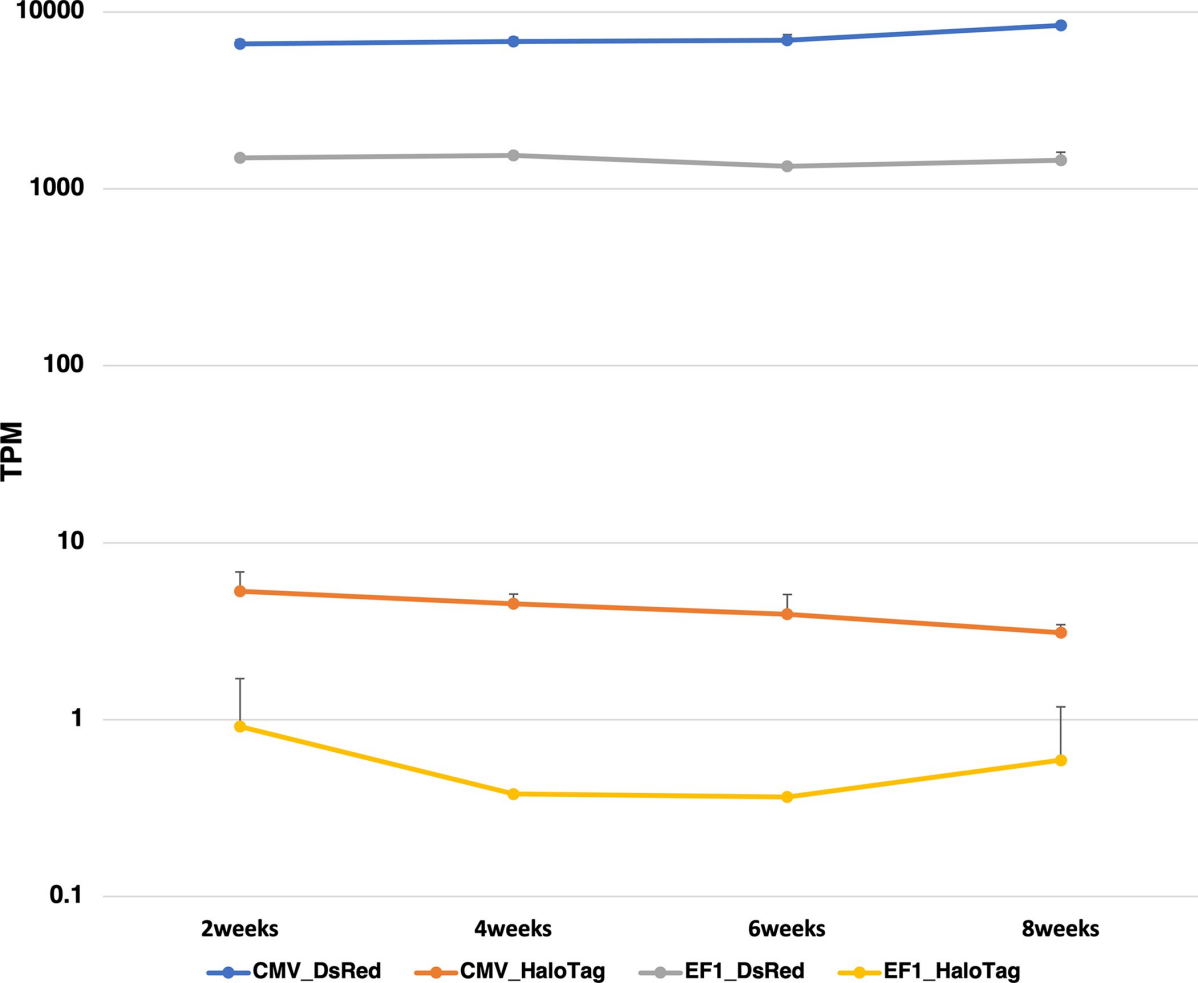
Transcripts per million (TPM) of CMV and EF1, DsRed, and Halo at 2, 4, 6, and 8 weeks. The line graphs show the mean value; the error bars indicate SD.

We used the HaloTag Flexi NR plasmids to investigate their expression in the genes introduced to the cell by Halo, as all 48 NR genes were originally expressed in HEK293. In the first experiment, the HaloTag 48 NR genes were inserted between the Vlox and Slox sites of another plasmid (Fig 1A). Equal amounts of the 48 plasmids were mixed and transfected using the Scre-VCre recombinase plasmid into HEK293 cells carrying the dual RMCE cassette composed of the CMV- or EF1 promoter-driven DsRed gene at adeno-associated virus integration (AAVS) site 1, known as a safe harbor (Fig 1B). We confirmed the successful integration of a single copy of the dual RMCE cassette at the AAVS site 1 genomic location through PCR and an EGFP replacement experiment, following the methodology of Hasegawa et al. [12]. As the 48 NR plasmids lack promoters, gene expression only occurs following correct recombination. After 3 days of transfection, to correct the fluctuation of the 48 NR population during cell passage, the cells were divided into three equal volumes, and they were subsequently cultured separately. At 2 weeks after transfection, the expression of all 48 NRs was confirmed in both CMV- and EF1 promoter-driven cells by sequencing the HaloTag of the 48 NRs in at least one subdivided clone (S3 Table). The difference between the maximum and minimum expression levels among the 48 NR genes was 59-fold with EF1, but this was increased to 674-fold with CMV. Whole RNA-Seq analyses showed that the transcripts per million (TPM) value of CMV-DsRed was 6,598.5 and that of EF1 was 1,499.5 at 2 weeks after transfection (S4 Table). Both TPM values indicated high-expression levels within the respective cells; the values of CMV-DsRed were, in particular, the top 10 highest expression genes in the cell (S4 Table). The one-way ANOVA showed that both CMV- and EF1 promoter-driven expression of Halo, where the TPM value of Halo was the sum of all 48 NRs, continued expression at 2, 4, 6, and 8 weeks without significant changes (Fig 2). However, there was significant variation in the expression levels of the 48 individual NR genes driven by the CMV and EF1 promoters. As time progressed, among the genes that exhibited consistent expression patterns across all subclones at all time points, CMV-PPARG and EF1-NR1I2 showed increased relative levels; CMV-NR0B2 genes showed little change, and the expression levels of all three subclones of 16 NRs (CMV-ESRRA, CMV-ESRRG, CMV-NR2C2, CMV-NR2F2, CMV-NR5A1, CMV-NR5A2, EF1-ESR1, EF1-ESRRA, EF1-ESRRG, EF1-NR2C2, EF1-NR2E1, EF1-NR3C2, EF1-NR5A1, EF1-NR5A2, EF1-NR6A1, and EF1-RARA) disappeared at 8 weeks (S1 Fig). In the competition experiments of CMV- or EF1 promoter-driven 48 NRs, EF1-NR1I2 exhibited the largest increase from 2 to 8 weeks, whereas EF1-NR4A1 showed the maximum decrease at 8 weeks. EF1-RORA showed a relatively small TPM value of 7,153 at 2 weeks and then decreased rapidly (S3 Table).

### PCR-tagged gene insertion

Equimolar proportions of all 48 NRs were transfected using the Scre-VCre expression plasmid in HEK293 EF1-AAVS cells. Ten days after transfection, the expression of all 48 NRs was confirmed via sequencing of the HaloTag 48 NR libraries (S3 Table). The maximum gene expression level of NR5A1 was 1,271-fold higher than the minimum expression level of AR.

### Proliferation of stable NR-expressing cell lines

**Fig 3:**
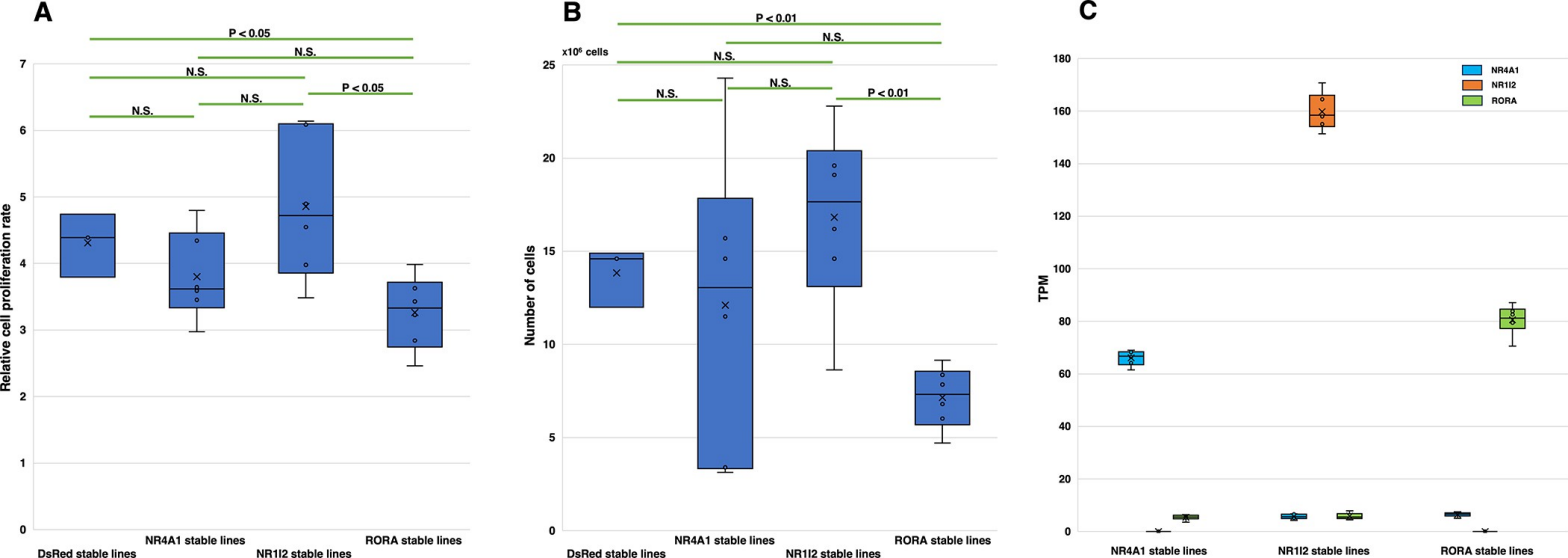
Characteristics of EF1-driven single copy NR1I2, NR4A1, or RORA stably-transfected cells. A. Box plots depicting the relative cell proliferation rates. Amount of increase after 64 h of culture. The P-values of Welch’s t-test are shown. B. Box plots illustrating the cell counts after 63 h of culture (seeding density: 5 ×10^5^ cells). The P-values of Welch’s t-test are shown. C. Box plots showing the TPM of the NR4A1, NR1I2, or RORA genes in stable lines.

We confirmed the insertion of the NR gene via dual RMCE using PCR and DNA sequencing. After 2.7 days of culture, the proliferation rate of the EF1 promoter-driven single copy of the NR1I2 gene line was 1.13-fold higher than that of the DsRed line (Fig 3A), as determined using the luminescent cell viability assay. On the other hand, the RORA line showed the lowest proliferation rate at about three-quarters of that of the DsRed line. The proliferation rate of the EF1-NR4A1 was intermediate and 0.88 times slower than that of the DsRed line. The results of the cell counting assay were consistent with those of the luminescent cell assay. After 2.6 days of culture, the total cell number of the EF1 promoter-driven single-copy NR1I2 gene line was 1.22-fold higher than that of the DsRed line (Fig 3B).

The RNA-Seq analyses revealed that all six clonal lines in each stable NR group expressed similar levels of the inserted NR transgene, which was more than tenfold higher than that of the other two stable NR groups (Fig 3C). In contrast, the TPM value of Halo showed small variations among the three NR stable lines. The mean value of NR4A1, NR1I2, and RORA was, 478.8, 570.4 and 665.3, respectively.

## Discussion

To accurately compare the effects of transgenes in inserted cells, it is necessary to express all genes at the same level. However, this is challenging to achieve with gene expression methods using plasmids, transposons, and virus-based vectors as expression from the same copies of transgenes in all transfected cells is desired. In addition, for studying CC [2], it is necessary to insert multiple genes simultaneously. In this study, we utilized KDRI HaloTag 48 NR clones to achieve expression of a single-copy gene within individual cells. We then examined the effects of elevated NR expression levels on the cell proliferation rate in immortalized human embryonic kidney (HEK293) cells. As our dual RMCE cassettes include a promoter and the NR48 genes used are promoterless (Fig 1B), we ensure that only correctly integrated genes are expressed. This results in the expression of a single-copy gene in each cell. The experimental conditions described in this paper closely mimic the conditions of early-stage cancer, characterized by numerous *de novo* mutations within a relatively small population compared to the surrounding normal cells. Our system is expected to be useful in investigating the cancer growth.

The expression level of CMV-driven DsRed was approximately 5-fold higher than that of the EF1 promoter-driven counterpart during the 8 weeks of competition (Fig 2). Both DsRed values exhibited very high-expression levels within their respective cells; in particular, the values of CMV-driven DsRed were among the top 10 highest expression genes in the cell (S4 Table). NRs inserted via dual RMCE must be expressed at the same expression levels as the DsRed genes within the cell. It is natural that the states of the 48 NRs during CC differed between CMV and EF1 promoters.

Of the 48 NRs, the gene expression of all subclones of 6 CMV-driven NRs and 10 EF1 promoter-driven NRs disappeared at 8 weeks. This finding could be attributed to multiple interrelated factors: competition with other NRs, competition with DsRed, and accidents during cell passaging. Although many samples showed low TPM values at 2 weeks, it is also possible that a rapid decrease in TPM value occurred within the first 2 weeks. In fact, the stable cell line of EF1-RORA showed very slow proliferation (Fig 3A and 3B). In particular, the strong overexpression of 5 NRs (ESRRA, ESRRG, NR2C2, NR5A1, and NR5A2), which disappeared in both the CMV- and EF1 promoter-driven cells, could have resulted in reduced or ceased proliferation. It has been reported that ESRRG overexpression abrogates cell growth and metastasis *in vitro* and *in vivo* [20]. In the competition experiments, EF1-NR1I2 showed the highest increase in the TPM values from 2 to 8 weeks, whereas EF1-NR4A1 exhibited the maximum decrease. In the stable cell line experiments using the luminescent cell viability assay, the cell proliferation rate of EF1-NR1I2 was 1.28- and 1.13-fold higher than that of EF1-NR4A1 and EF1-DsRed, respectively. Irrespective of the normalization method used, comparing the absolute expression levels among different genes is challenging owing to differences in their lengths not only among clones but also within a cell. However, this comparison becomes feasible for HaloTag genes. The expression levels of Halo in stable NR4A1, NR1I2 and RORA lines were within 1.4-fold. In our transgene competition system, the expression levels of the inserted genes were similar among stable lines of different NR genes. These results show that the overexpression of an NR gene from a single-copy gene in one cell had a significant effect on cell growth and that genes for a particular purpose could be screened using the CC experiment with our transgene competition system.

To expand the application of this system, we have been proceeding with various means. In this study, we used the PCR-tagged method to express all 48 NRs. The thymidine kinase (TK) gene has been added to the cassette for negative selection of the EF1 or CMV-Vlox-DsRed-TK-Slox cassette. These cassettes are useful for not only transgene competition but also stable cell line production. The transgene competition system described herein has already been loaded on the human artificial chromosome (HAC) vector [12]. The HAC vector is an episomal vector and can accommodate DNA inserts of any size [21]. As HAC can be transferred from any host cell to any recipient cell by isolated metaphase chromosome transfection or microcell mediated chromosome transfer [12,21–23], our system can be utilized in desired cells rapidly. In addition, there are over 10,000 HaloTag clones and over 12,000 cDNA clones, including 640 GPCRs, 930 transcription factors, and 800 kinases in the Kazusa collection (http://www.kazusa.or.jp/kop/dsearch/, https://www.promega.jp/products/pm/halotag-technology/kazusa-collection/?activeTab=1#tab-2). The key advantage of the Kazusa clone is that the majority possess a HaloTag clone. Combining our new system with the Kazusa collection would enable selective gene expression for various applications.

## Supporting information

S1 Fig Time course changes in gene expression of each nuclear receptor. (TIFF)

S1 Table KDRI 48 NRs clone list. (XLSX)

S2 Table Primers and PCR conditions. (DOCX)

S3 Table TPM values of the 48 nuclear receptors at 2, 4, 6, and 8 weeks and TPM values of PCR-tagged gene insertion at 10 days. (XLSX)

S4 Table TPM values of whole RNA-Seq at 2, 4, 6, and 8 weeks. (XLSX)
