# Supplementary figures and images for: A cell competition system with one gene expression from a single-copy gene in one cell
Source: PLoS One. 2024 Jul 5;19(7):e0302451. doi: 10.1371/journal.pone.0302451 (PMC11226009; doi:10.1371/journal.pone.0302451)

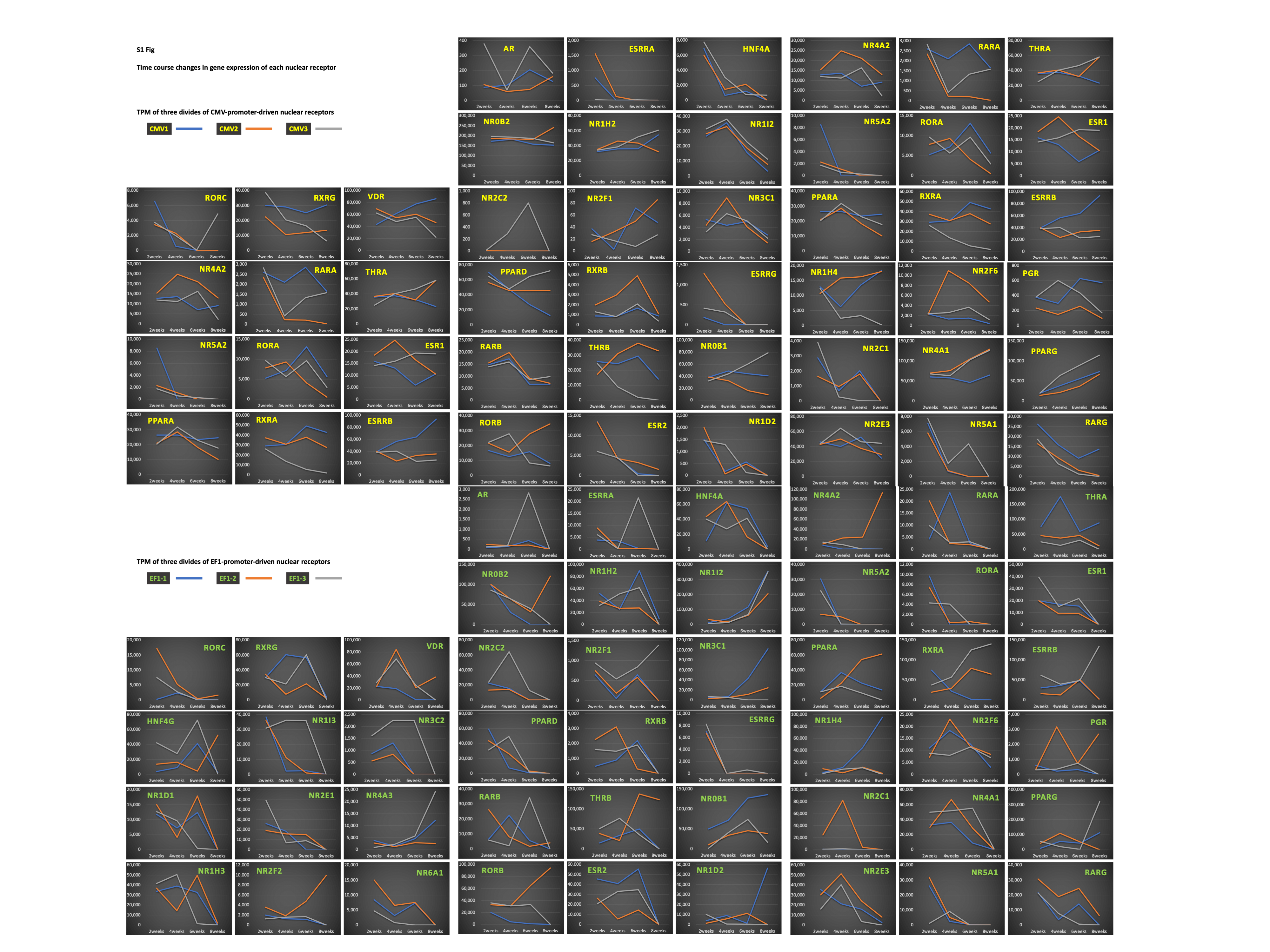

Supplement: S1 Fig — (TIFF) [file pone.0302451.s001.tiff]
