# Supplementary material for: A cell competition system with one gene expression from a single-copy gene in one cell
Source: PLoS One. 2024 Jul 5;19(7):e0302451. doi: 10.1371/journal.pone.0302451 (PMC11226009; doi:10.1371/journal.pone.0302451)
Supplement: S2 Table — (DOCX) [file pone.0302451.s003.docx]

**S2 Table**

**Primers and PCR conditions**

(1). Halo tag gene PCR

cDNA 50ng

i). 1st PCR

PrimeSTAR GXL DNA Polymerase (TAKARA, Japan)

Primer

Final 300nM, Halo-1^st^

TCGGAAATTGAGCCACCATG

Final 300nM, pFN21A_PmeI_1^st^

CTTTCGGGCTTTGTTAGCAG

PCR condition

95 °C 1 min

(98 °C 10sec, 60°C 15sec, 68°C 45sec) x21cycle

ii). 2nd nested PCR

PrimeSTAR GXL DNA Polymerase (TAKARA, Japan)

Primer

Final 300nM, Halo-2^nd^

ATCGGTACTGGCTTTCCATTCG

Final 300nM, pFN21A_PmeI_2nd-M13-R

CCTGCAGGTCGACTCTAGAG

PCR condition

95 °C 1 min

(98 °C 10sec, 60°C 15sec, 68°C 90sec) x16cycle

iii). NGS library preparation

NEBNext Ultra II FS DNA Library Prep Kit for Illumina according to the manufacturer’s instructions

5cycles

(2). Vlox Slox tagged PCR

i). 1st PCR

Ex Premier (TAKARA, Japan)

Primer

Final 200nM, Vlox_Halo_L CGTCGGTCCCGGCATCCGATACTAGTTCAATTTCTGAGAACTGTCATTCTCGGAAATTGAGCCACCATGGCAGAAATCGG

Final 200nM, polyA_Slox_R1_3_2022

CTCCTCGGCCACGAAGTGCACTAGTCTCGTGTCCGATAATTACAGTTATCGGACACGAGCGCAAGCGCAAAGAGGATCC

PCR condition

94°C 1 min

(98°C 10sec, 60°C 15sec, 68°C 3mins) x30cycle

ii). 2nd PCR

Ex Premier (TAKARA, Japan)

Primer

Final 200nM, Vlox_Halo_S_5S_2022

CGTCGGTCCCGGCATCCGAT

Final 200nM, polyA_Slox_R2_5S_2022

CTCCTCGGCCACGAAGTGCA

PCR condition

94°C 1 min

(98°C 10sec, 60°C 15sec, 68°C 3mins) x30cycle

Agarose gel electrophoresis of two-step PCR product


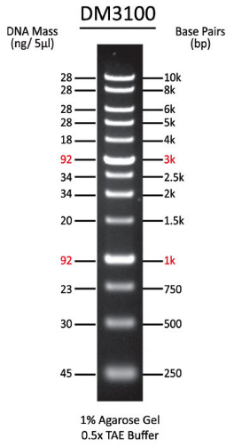


| **lane01** | **1kb ladder** |  |  |  |
| --- | --- | --- | --- | --- |
| **lane02** | **NR_No.01** | **NR0B1** | **KIBB7043** | **2912 bp** |
| **lane03** | **NR_No.02** | **NR1H2** | **KIBB8155** | **2882 bp** |
| **lane04** | **NR_No.01** | **NR0B1** | **KIBB7043** | **2912 bp** |
| **lane05** | **NR_No.02** | **NR1H2** | **KIBB8155** | **2882 bp** |
| **lane06** | **NR_No.01** | **NR0B1** | **KIBB7043** | **2912 bp** |
| **lane07** | **NR_No.02** | **NR1H2** | **KIBB8155** | **2882 bp** |
| **lane08** | **1kb ladder** |  |  |  |


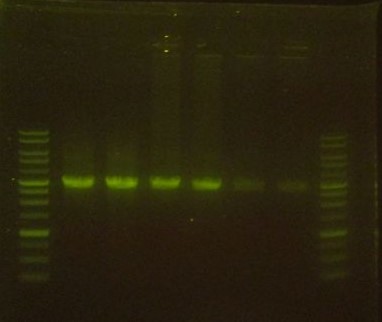


1 2 3 4 5 6 7 8


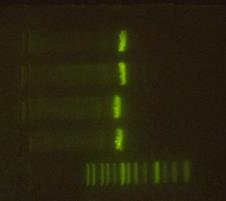


| **lane01** | **1kb ladder** |  |  |  |
| --- | --- | --- | --- | --- |
| **lane02** | **NR_No.03** | **RXRB** | **KIBB4058** | **3113 bp** |
| **lane03** | **NR_No.04** | **NR4A3** | **KIBB3311** | **3380 bp** |
| **lane04** | **NR_No.05** | **PPARD** | **KIBB4629** | **2825 bp** |
| **lane05** | **NR_No.06** | **VDR** | **KIBB7719** | **2783 bp** |

1 2 3 4 5


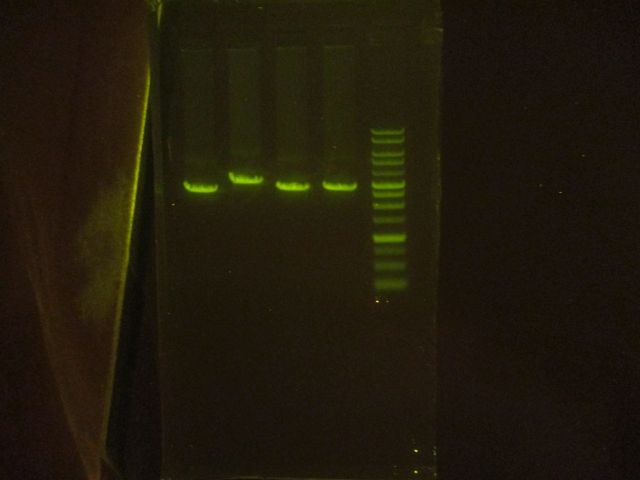


| **lane01** | **1kb ladder** |  |  |  |
| --- | --- | --- | --- | --- |
| **lane02** | **NR_No.07** | **NR1H3** | **KIBB7759** | **2843 bp** |
| **lane03** | **NR_No.08** | **NR1D1** | **KIBB7838** | **3344 bp** |
| **lane04** | **NR_No.09** | **RXRG** | **KIBB5869** | **2891 bp** |
| **lane05** | **NR_No.11** | **NR6A1** | **KIEE3212** | **2939 bp** |

1 2 3 4 5


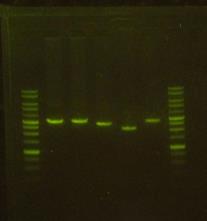


| **lane01** | **1kb ladder** |  |  |  |
| --- | --- | --- | --- | --- |
| **lane02** | **NR_No.12** | **ESR2** | **KIBB5914** | **3092 bp** |
| **lane03** | **NR_No.13** | **RORC** | **KIEE3637** | **3056 bp** |
| **lane04** | **NR_No.14** | **NR2E3** | **KIBB7686** | **2732 bp** |
| **lane05** | **NR_No.15** | **NR0B2** | **KIBB8915** | **2273 bp** |
| **lane06** | **NR_No.16** | **THRA** | **KIBB7752** | **2972 bp** |
| **lane07** | **1kb ladder** |  |  |  |

1234567


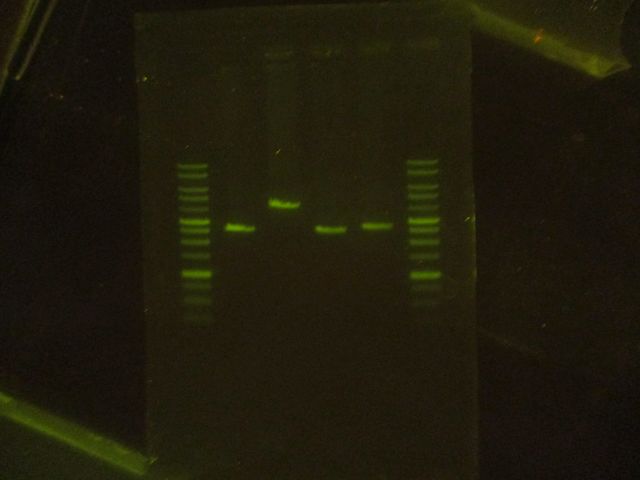


| **lane01** | **1kb ladder** |  |  |  |
| --- | --- | --- | --- | --- |
| **lane02** | **NR_No.17** | **NR2E1** | **KIBB4185** | **2657 bp** |
| **lane03** | **NR_No.18** | **AR** | **KIEE1197** | **4253 bp** |
| **lane04** | **NR_No.19** | **NR1I3** | **KIBB7608** | **2573 bp** |
| **lane05** | **NR_No.20** | **NR2F1** | **KIBB8385** | **2771 bp** |
| **lane06** | **1kb ladder** |  |  |  |

123456


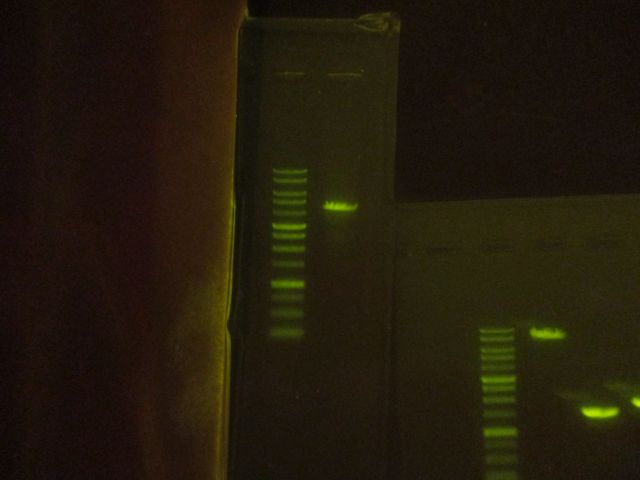


| **lane01** | **1kb ladder** |  |  |  |
| --- | --- | --- | --- | --- |
| **lane02** | **NR_No.10** | **NR3C2** | **KIBB0468** | **4454 bp** |

12


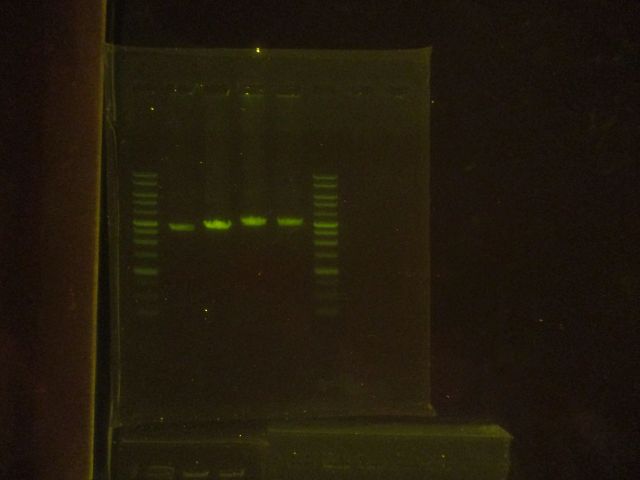


| **lane01** | **1kb ladder** |  |  |  |
| --- | --- | --- | --- | --- |
| **lane02** | **NR_No.21** | **HNF4A** | **KIBB9731** | **2924 bp** |
| **lane03** | **NR_No.22** | **RXRA** | **KIBB9770** | **2888 bp** |
| **lane04** | **NR_No.23** | **NR2C2** | **KIBB5580** | **3347 bp** |
| **lane05** | **NR_No.24** | **ESR1** | **KIBB7810** | **3287 bp** |
| **lane06** | **1kb ladder** |  |  |  |

123456


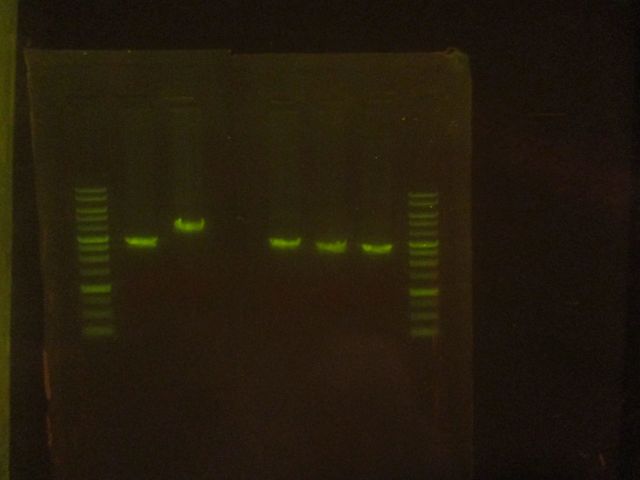


| **lane01** | **1kb ladder** |  |  |  |
| --- | --- | --- | --- | --- |
| **lane02** | **NR_No.25** | **NR2F2** | **KIBB7696** | **2744 bp** |
| **lane03** | **NR_No.26** | **NR3C1** | **KIBB9466** | **3833 bp** |

123


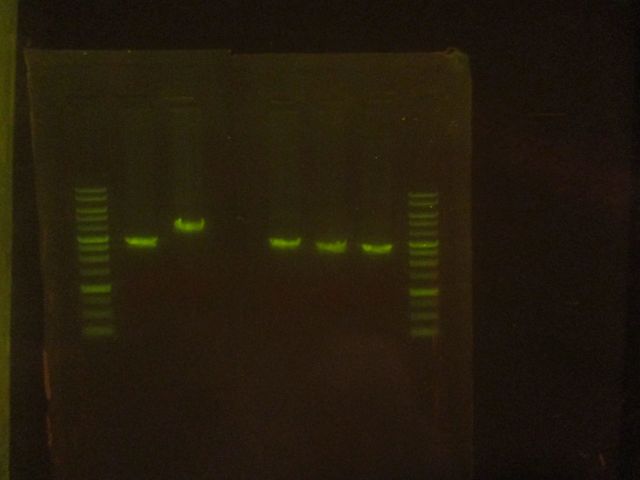


| **lane01** | **NR_No.27** | **NR1D2** | **KIBB9131** | **3239 bp** |
| --- | --- | --- | --- | --- |
| **lane02** | **NR_No.28** | **NR1I2** | **KIBB7714** | **2921 bp** |
| **lane03** | **NR_No.29** | **ESRRA** | **KIBB7713** | **2771 bp** |
| **lane04** | **1kb ladder** |  |  |  |

1234


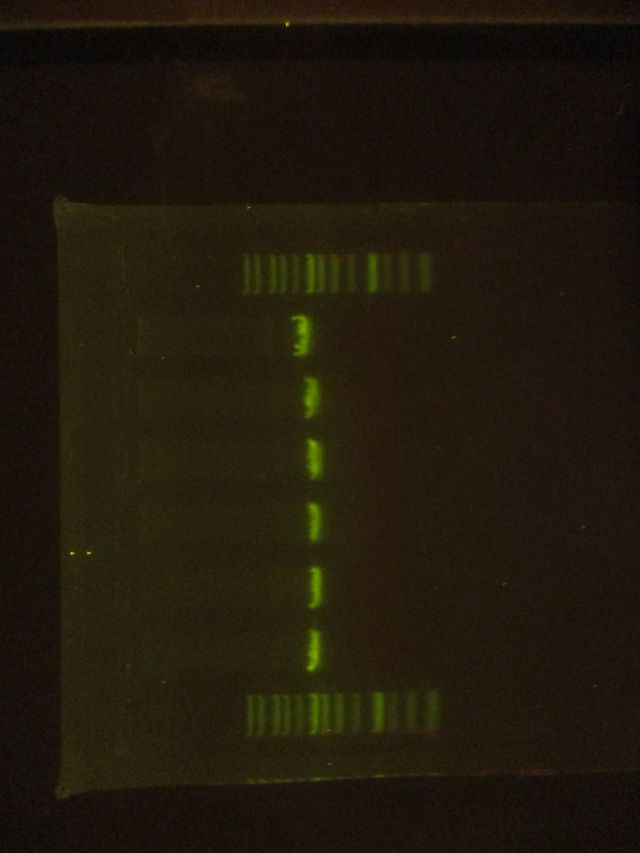


| **lane01** | **1kb ladder** |  |  |  |
| --- | --- | --- | --- | --- |
| **lane02** | **NR_No.30** | **PPARG** | **KIBB7770** | **2933 bp** |
| **lane03** | **NR_No.31** | **RORB** | **KIBE0940** | **2879 bp** |
| **lane04** | **NR_No.32** | **RARA** | **KIBE1591** | **2888 bp** |
| **lane05** | **NR_No.33** | **RARB** | **KIBB5810** | **2846 bp** |
| **lane06** | **NR_No.34** | **NR1H4** | **KIBB7740** | **2948 bp** |
| **lane07** | **NR_No.35** | **NR2C1** | **KIBB6166** | **3311 bp** |
| **lane08** | **1kb ladder** |  |  |  |

12345678


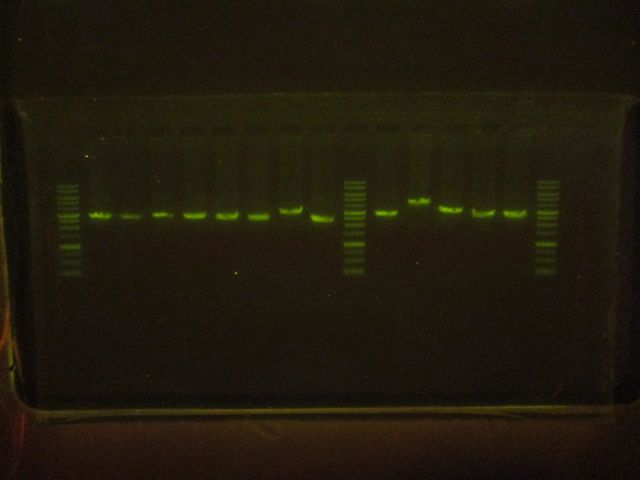


| 1 | 2 | 3 | 4 | 5 | 6 | 7 | 8 | 9 | 10 | 11 | 12 | 13 | 14 | 15 | 16 |
| --- | --- | --- | --- | --- | --- | --- | --- | --- | --- | --- | --- | --- | --- | --- | --- |

| **lane01** | **1kb ladder** |  |  |  |
| --- | --- | --- | --- | --- |
| **lane02** | **NR_No.36** | **NR5A1** | **KIBB7702** | **2885 bp** |
| **lane03** | **NR_No.37** | **NR5A2** | **KIBB5946** | **2987 bp** |
| **lane04** | **NR_No.38** | **ESRRB** | **KIBB9737** | **3002 bp** |
| **lane05** | **NR_No.39** | **ESRRG** | **KIAA0832** | **2876 bp** |
| **lane06** | **NR_No.40** | **THRB** | **KIBB3732** | **2885 bp** |
| **lane07** | **NR_No.41** | **NR2F6** | **KIBB9720** | **2714 bp** |
| **lane08** | **NR_No.42** | **NR4A2** | **KIBB7818** | **3296 bp** |
| **lane09** | **NR_No.43** | **NR4A1** | **KIBB9634** | **2477 bp** |
| **lane10** | **1kb ladder** |  |  |  |
| **lane11** | **NR_No.44** | **PPARA** | **KIBB9549** | **2906 bp** |
| **lane12** | **NR_No.45** | **PGR** | **KIBB9766** | **4301 bp** |
| **lane13** | **NR_No.46** | **RORA** | **KIBB5968** | **3170 bp** |
| **lane14** | **NR_No.47** | **RARG** | **KIBB5808** | **2864 bp** |
| **lane15** | **NR_No.48** | **HNF4G** | **KIBB7751** | **2837 bp** |
| **lane16** | **1kb ladder** |  |  |  |
